# Cell Type-Specific Predictive Models Perform Prioritization of Genes and Gene Sets Associated With Autism

**DOI:** 10.3389/fgene.2020.628539

**Published:** 2021-01-15

**Authors:** Jinting Guan, Yang Wang, Yiping Lin, Qingyang Yin, Yibo Zhuang, Guoli Ji

**Affiliations:** ^1^Department of Automation, Xiamen University, Xiamen, China; ^2^National Institute for Data Science in Health and Medicine, Xiamen University, Xiamen, China; ^3^Xiamen YLZ Yihui Technology Co., Ltd., Xiamen, China; ^4^Innovation Center for Cell Signaling Network, Xiamen University, Xiamen, China

**Keywords:** autism spectrum disorder, cell type-specific, predictive model, gene set, biomarker

## Abstract

Bulk transcriptomic analyses of autism spectrum disorder (ASD) have revealed dysregulated pathways, while the brain cell type-specific molecular pathology of ASD still needs to be studied. Machine learning-based studies can be conducted for ASD, prioritizing high-confidence gene candidates and promoting the design of effective interventions. Using human brain nucleus gene expression of ASD and controls, we construct cell type-specific predictive models for ASD based on individual genes and gene sets, respectively, to screen cell type-specific ASD-associated genes and gene sets. These two kinds of predictive models can predict the diagnosis of a nucleus with known cell type. Then, we construct a multi-label predictive model for predicting the cell type and diagnosis of a nucleus at the same time. Our findings suggest that layer 2/3 and layer 4 excitatory neurons, layer 5/6 cortico-cortical projection neurons, parvalbumin interneurons, and protoplasmic astrocytes are preferentially affected in ASD. The functions of genes with predictive power for ASD are different and the top important genes are distinct across different cells, highlighting the cell-type heterogeneity of ASD. The constructed predictive models can promote the diagnosis of ASD, and the prioritized cell type-specific ASD-associated genes and gene sets may be used as potential biomarkers of ASD.

## Introduction

Autism spectrum disorder (ASD) represents a group of neurodevelopmental disorders, characterized by substantial phenotypic and genetic heterogeneity. Genetic studies have identified variants that contribute to the risk of developing ASD (Iossifov et al., 2012; Neale et al., 2012; O’Roak et al., 2012; Sanders et al., 2012; De Rubeis et al., 2014; Gaugler et al., 2014; Turner et al., 2016; Satterstrom et al., 2020). However, it remains perplexing how these reported variants lead to the pathogenesis of ASD. A major mode of action is that these genetic variants cause gene expression alternations; direct analysis of gene expression in disease-relevant tissue is thus valuable for understanding the molecular mechanism of ASD. As ASD is believed to result from functional aberrations within brains, bulk transcriptomic analyses between autistic and normal brains have been applied for identifying aberrant gene expression patterns in ASD (Voineagu et al., 2011; Gupta et al., 2014; Guan et al., 2016; Parikshak et al., 2016). However, the brain is a highly heterogeneous organ including different cell types that are highly interconnected. Genes may demonstrate diverse functions across different brain cell types. In ASD, different functions may be dysregulated and causal genes may be distinct across different cells. Although bulk transcriptomic studies revealed convergence of disease pathology on common pathways, the brain cell type-specific molecular pathology of ASD is still needed to study.

Recently, the newly available single-nucleus RNA-sequencing data of ASD (Velmeshev et al., 2019) makes it possible to study the cell-type heterogeneity of ASD directly. The authors identified differentially expressed (DE) genes between ASD and control groups in a cell type-specific way and analyzed the functions of the cell type-specific DE genes to characterize the heterogeneity of dysregulated gene expression patterns among brain cell types in ASD. As genes interact with others, the integrity of disease gene modules instead of individual genes may determine the manifestation of a disease in cells (Kitsak et al., 2016; Mohammadi et al., 2019). Therefore, in addition to identifying the individual cell type-specific risk genes, it is essential to identify cell type-specific gene sets/modules associated with ASD.

There have been more and more studies evaluating the effectiveness of machine learning for diagnosing ASD, exploring its genetic underpinnings, and designing effective interventions (Hyde et al., 2019). These studies were based on different kinds of datasets, such as behavior evaluation based on Autism Diagnostic Observation Schedule (ADOS) (Duda et al., 2014; Levy et al., 2017) and Autism Diagnostic Interview-Revised (ADI-R) (Wall et al., 2012; Duda et al., 2016), brain images for magnetic resonance image (MRI) (Chen et al., 2011; Heinsfeld et al., 2018) and electroencephalogram (EEG) (Bosl et al., 2018), and genetic profiles (Kong et al., 2012; Cogill and Wang, 2016; Guan et al., 2016; Oh et al., 2017). To detect ASD candidate genes, several predictive models were constructed based on gene expression profiling, including the one built using DE genes between ASD and controls based on gene expression microarrays of blood (Kong et al., 2012) and the one built using aberrant gene expression in ASD based on bulk transcriptomic data of brains (Guan et al., 2016). Actually, for identifying ASD risk genes, genetic and genomic studies were usually performed, such as genome-wide association studies, copy number variation studies, and whole exome sequencing; these methods are expensive and time-consuming, and the generated potential candidate genes are numerous and not easy to be validated (Cogill and Wang, 2016). Gene screening methods based on machine learning can prioritize genes and identify high-confidence candidates, which may provide new insights for the experimental studies.

In this study, to characterize the cell-type heterogeneity of ASD and to take advantage of the potential of gene expression signature being diagnostic biomarkers for ASD, we analyze the human brain nucleus gene expression data of ASD and controls published in Velmeshev et al. (2019) and construct multiple kinds of classification models for ASD using the algorithm of partial least squares (PLS), identifying cell type-specific genes and gene sets associated with ASD. Firstly, we construct cell type-specific predictive models based on individual genes to screen cell type-specific genes associated with ASD. Then, we construct cell type-specific gene set-based predictive models to screen cell type-specific gene sets associated with ASD. These two kinds of predictive models can be applied to predict the diagnosis of a given nucleus with known cell type. Lastly, we further construct a multi-label predictive model for predicting the cell type and diagnosis of a given nucleus at the same time. Our results suggest that it may be feasible to use brain cell/nucleus gene expression for ASD detection and the constructed predictive models can promote the diagnosis of ASD. Our analytical pipeline prioritizes ASD-associated cell type-specific genes and gene sets, highlighting the cell-type heterogeneity of ASD.

## Materials and Methods

### Human Brain Nucleus Gene Expression Data

We used the single-nucleus RNA-seq data published in Velmeshev et al. (2019), which includes 104,559 nuclei from 41 post-mortem tissue samples from the prefrontal cortex and anterior cingulate cortex of 15 ASD patients and 16 control subjects. The nuclei were divided into 17 cell types, including fibrous astrocytes (AST-FB), protoplasmic astrocytes (AST-PP), endothelial, parvalbumin interneurons (IN-PV), somatostatin interneurons (IN-SST), SV2C interneurons (IN-SV2C), VIP interneurons (IN-VIP), layer 2/3 excitatory neurons (L2/3), layer 4 excitatory neurons (L4), layer 5/6 corticofugal projection neurons (L5/6), layer 5/6 cortico-cortical projection neurons (L5/6-CC), microglia, maturing neurons (Neu-mat), NRGN-expressing neurons I (Neu-NRGN-I), NRGN-expressing neurons II (Neu-NRGN-II), oligodendrocytes, and OPC. We downloaded the matrices of raw counts from the website of autism.cells.ucsc.edu. Then, we preprocessed the data with R package of scran (Lun et al., 2016), including the quality control of nuclei and genes, removing a minority of nuclei from different cell cycle phases, and normalizing the gene expression data. Next, nuclear and mitochondrial genes downloaded from Human MitoCarta2.0 (Calvo et al., 2016) were excluded. We used the function of plotExplanatoryVariables in scran to check if any factors, including region, age, sex, PMI (post-mortem interval), RIN (RNA integrity number), Capbatch (10X capture batch), and Seqbatch (sequencing batch), may contribute to the heterogeneity of gene expression. It can calculate the percentage of the variance of the expression values that is explained by the factors for each gene. By checking the distribution of percentages across all genes, we found that the expression profiles of most genes are not strongly associated with the factors and the factors thus do not need to be explicitly modeled in the downstream analyses (Lun et al., 2016). We applied scran to obtain highly variable genes, which include a total of 12,036 genes. We used the expression level of 12,036 genes for downstream analyses, which contains 85,125 nuclei, including 3655, 7085, 1991, 3719, 4190, 1836, 5621, 12,795, 6518, 3402, 4385, 2495, 3532, 589, 1459, 12206, and 9647 nuclei from cell types of AST-FB, AST-PP, endothelial, IN-PV, IN-SST, IN-SV2C, IN-VIP, L2/3, L4, L5/6, L5/6-CC, microglia, Neu-mat, Neu-NRGN-I, Neu-NRGN-II, oligodendrocytes, and OPC, respectively.

### Annotated Gene Sets

A total of 913 ASD candidate genes were downloaded from Simons Foundation Autism Research Initiative (SFARI) (release of March 4, 2020), which include 119, 144, 219, and 472 genes from categories of S (syndromic), 1 (high confidence), 2 (strong candidate), and 3 (suggestive evidence). For gene set analysis, three kinds of annotated gene sets from Molecular Signatures Database (MSigDB) (Liberzon et al., 2011) were used, including H: hallmark gene sets, C2: curated gene sets (containing gene sets from chemical and genetic perturbations, and canonical pathways of Biocarta, KEGG, PID, and Reactome), and C5: GO gene sets. By intersecting the genes in gene sets and our analyzed gene expression matrix, we kept 3741 gene sets containing more than 30 overlapping genes.

### The Algorithm of Partial Least Squares

Partial least squares (Wold, 1966) regression combines features from principal component analysis and multiple regression. It has the ability to address the problem of modeling multicollinearity, noisy, and even incomplete highly dimensional data (Boulesteix and Strimmer, 2006). PLS can solve both single- and multi-label classification problems. Partial least squares discriminant analysis (PLS-DA) is a PLS regression, with the dependent variable being categorical. Suppose *X* is an *n* × *m* matrix containing *n* observations of *m* genes and *Y* is an *n* × *p* matrix containing *n* observations of *p* response variables, then *X* and *Y* can be decomposed by:

$$X=T\,P^{T}+E,Y=U\,Q^{T}+F$$

where *T* and *U* are *n* × *k* score matrices (called component scores or latent variables) of *X* and *Y*, respectively, *P* and *Q* are *m* × *k* and *p* × *k* orthogonal loading matrices, and *E* and *F* are the residual matrices. The decompositions of *X* and *Y* are made so as to maximize the covariance between *T* and *U*. Then, based on *T*, *P*, *U*, and *Q*, we can first fit *U* and *T*, and then the linear relationship between *X* and *Y* can be obtained.

### Recursive Feature Elimination With Cross-Validation

Recursive feature elimination (RFE) (Guyon et al., 2002) is a backward feature selection method, which is a recursive process. It first builds a model using all features based on an algorithm specified, such as PLS in our study, and computes a measure of importance for each feature. The least important features are removed. Then, the model is re-built using the left features, importance scores are computed, and the least important features are removed until the specified number of features is reached. RFE attempts to eliminate dependencies and collinearity that may exist in the model. It requires a specified number of features to keep. To find the optimal number of features, RFE with cross-validation (RFECV) is usually used to score feature subsets of different sizes and select the best scoring one. Then, the optimal feature subset is used to build the final model.

### The Construction of Predictive Models

The R package of caret (Kuhn, 2008) was adopted to construct predictive models based on the algorithm of PLS. Firstly, for each cell type, we extracted the gene expression data of nuclei from the cell type and constructed a cell type-specific predictive model. Secondly, for each cell type and each annotated gene set, we extracted the expression data of nuclei from the cell type in the genes included in the gene set and constructed a cell type-specific gene set-based predictive model. These two kinds of predictive models can predict the diagnosis of a nucleus with known cell type. Specifically, we split the extracted gene expression data into a training set and a test set at a ratio of 7:3 using stratified sampling. For the training set, we selected the optimal model by applying 10-fold cross-validation for 10 times and tuning over the model hyperparameter (the number of PLS components) with grid search from 1 to 15 with a step of 1. To evaluate the model performance, the area under the receiver operating characteristic (ROC) curve (denoted as AUC) was used, because this metric can deal well with the problem of label imbalance and not be influenced by the selection of threshold. Then, from the optimal model, we obtained the predictive probability of each nucleus being a nucleus from ASD patients. Next, we used R package of pROC (Robin et al., 2011) to obtain the best threshold on training set and the threshold was used to determine the predictive performances on training set and test set. For each predictive model, we calculated the importance of each gene using the function of *varImp* in caret.

In order to predict the cell type and diagnosis of a given nucleus at the same time, we constructed a multi-label predictive model based on PLS using R package of mlr (Bischl et al., 2016). For each nucleus, we used 18 labels to describe it, with 1 label being the diagnosis and the other 17 cell-type labels obtained using one-hot encoding. We split the whole gene expression data including all cell types and all genes into a training set and a test set at a ratio of 7:3 using stratified sampling. Based on the training set, we selected the optimal model by applying five-fold cross-validation for five times and tuning over the model hyperparameter with grid search from 1 to 15 with a step of 1. Hamming loss, which is the fraction of labels that are predicted incorrectly to the total number of labels, was used as a performance indicator. Then, from the optimal model, we obtained the predictive probability of each nucleus belonging to each label. For the labels of cell types, the predictive cell type of each nucleus was set as the cell type whose predictive probability is the largest. For the diagnosis label, we extracted the predictive probability of training set and applied ROC analysis to obtain the optimal cut-off on training set for determining the predictive diagnosis of each nucleus in training and test sets.

## Results

### Methodological Overview

After normalization, we used the function of plotExplanatoryVariables in scran (Lun et al., 2016) to calculate the percentage of the variance of the expression values that is explained by factors, including region, age, sex, PMI, RIN, Capbatch (10X capture batch), and Seqbatch (sequencing batch), for each gene (Supplementary Figure 1A). We found that the expression profiles of most genes are not strongly associated with the factors and the factors thus do not need to be explicitly modeled in the downstream analyses. Then, we obtained highly variable genes, a total of 12,036 genes, and used their expression level for downstream analyses. The density plot of the percentage of variance explained by each factor across highly variable genes can be seen in Supplementary Figure 1B.

**FIGURE 1:**
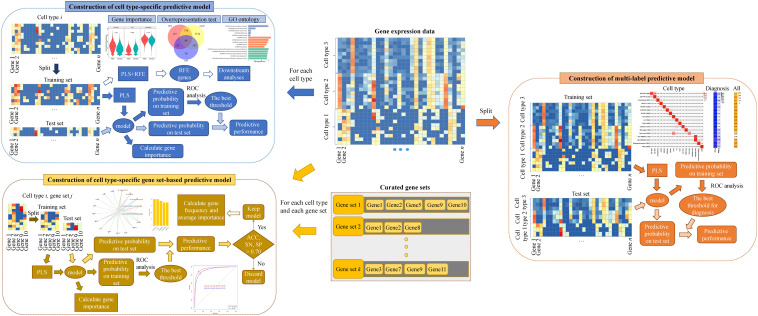
The *methodological overview*.

Then we constructed multiple kinds of predictive models for ASD. The overview of our analytical method can be seen in Figure 1. Firstly, to screen genes associated with ASD in each cell type, we constructed cell type-specific predictive models, which can predict the diagnosis of a nucleus whose cell type is known, using the algorithm of PLS (see section “Materials and Methods”). Specifically, for each cell type, we extracted the gene expression data of the nuclei from the cell type and split the data into training and test sets. We selected the optimal model based on the training set, and then obtained the predictive probability of each nucleus being a nucleus from ASD patients. Next, ROC analysis was performed to obtain the best threshold on training set, and the threshold was used to determine the predictive performance on training and test sets. To prioritize genes, we calculated the importance of each gene in the cell type-specific predictive model. In addition, in order to use less genes to achieve similar performances, we performed RFECV (see section “Materials and Methods”) to reduce the number of genes used to re-construct cell type-specific predictive models. The optimal genes obtained using RFECV were denoted as RFE genes, which were used for the downstream analyses to depict the cell-type heterogeneity of ASD.

Secondly, to screen gene sets associated with ASD in each cell type, we constructed cell type-specific gene set-based predictive models using PLS. Specifically, for each cell type and each gene set, we extracted the expression level of the nuclei from the cell type in the genes included in the considered gene set and constructed a predictive model. To prioritize gene sets, we ranked gene sets using their predictive performance on the test set and kept the gene sets whose predictive accuracy (ACC), sensitivity (SN), and specificity (SP) are larger than 70% as cell type-specific gene sets associated with ASD. Besides, for the total genes included in these identified gene sets, we calculated their frequency and averaged importance, and used the genes with top averaged importance to re-construct cell type-specific predictive models.

Lastly, we further constructed a multi-label predictive model using PLS, which can predict the cell type and the diagnosis of a given nuclei at the same time. For the labels of cell types, the predictive cell type of each nucleus was set as the cell type whose predictive probability is the largest. For the diagnosis label, we extracted the predictive probability of training set and applied ROC analysis to obtain the optimal cut-off for determining the predictive diagnosis of each nucleus in training and test sets.

### Cell Type-Specific Genes Associated With ASD

**FIGURE 2:**
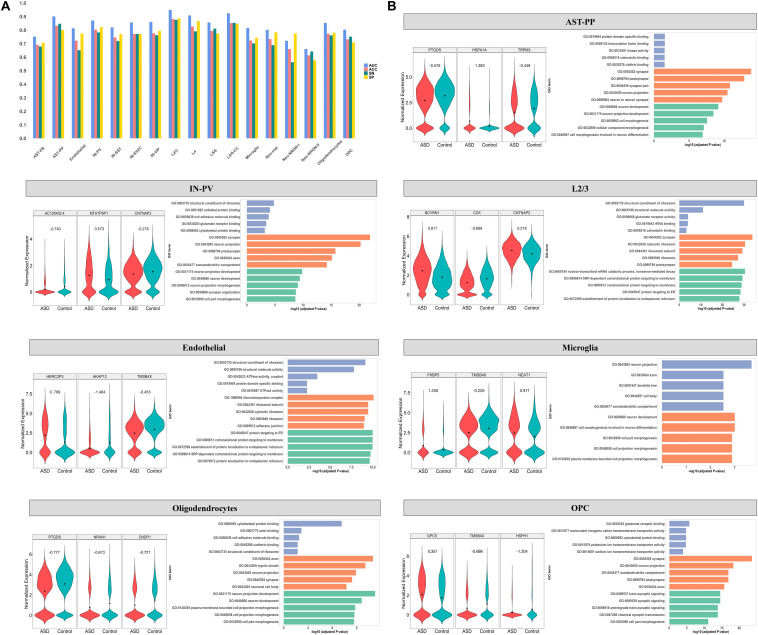
**(A)** The classification performance on test set of cell type-specific predictive models built using RFE genes. ROC analysis was applied to obtain the AUC and the optimal cut-off point on the training set, and then the optimal cut-off was used to determine the predictive accuracy (ACC), sensitivity (SN), and specificity (SP) on the test set. For the cell types of AST-PP, endothelial, IN-PV, L2/3, microglia, oligodendrocytes, and OPC, **(B)** the expression of the top three important genes in ASD and control groups is shown along with the top enriched GO terms with the RFE genes. The GO terms belonging to molecular functions, cellular component, and biological process are shown in blue, orange, and green, respectively.

For each of the 17 cell types, we first constructed a cell type-specific predictive model using all genes (Table 1 and Supplementary File 1). To score genes in each cell type, we calculated the importance of genes and ranked the genes (Supplementary File 2). Next, in order to use less genes to achieve similar performances, we used the genes with top 500, 1000, and 1500 importance respectively to construct cell type-specific predictive models. We found out that using top 1000 genes made the model performance better than the one using top 500, while approaching the one using top 1500 genes (Supplementary File 1). Therefore, for each cell type, we applied RFECV to reduce the number of genes to up to 1000 and obtain the optimal gene subset, which was then used to re-construct a cell type-specific predictive model (see section “Materials and Methods”). The R package of caret (Kuhn, 2008) was adopted to perform PLS-RFE with 10-fold cross-validation for 10 times. The sizes of evaluated gene subsets are from 100 to 1000 with a step of 100. The optimal genes obtained using RFECV were denoted as RFE genes. It is noted that the performances on test sets of the cell type-specific predictive models based on RFE genes approach the ones based on all genes (Figure 2A and Supplementary File 1); hence, we used the RFE genes for the subsequent analyses in this section.

**TABLE 1:** The classification performances of cell type-specific predictive models built using all genes.

Cell type (ASD/control)	Training set				Test set			
	ACC	SN	SP	AUC	ACC	SN	SP	AUC
AST-FB (2033/1622)	0.91	0.92	0.9	0.97	0.72	0.78	0.63	0.79
AST-PP (4749/2336)	0.93	0.93	0.93	0.98	0.84	0.87	0.79	0.90
Endothelial (850/1141)	0.92	0.91	0.92	0.97	0.76	0.70	0.80	0.83
IN-PV (1811/1908)	0.95	0.94	0.96	0.99	0.80	0.77	0.82	0.88
IN-SST (1945/2245)	0.94	0.92	0.95	0.98	0.76	0.70	0.81	0.83
IN-SV2C (990/846)	0.98	0.98	0.97	1.00	0.80	0.83	0.76	0.88
IN-VIP (3098/2523)	0.89	0.88	0.91	0.96	0.79	0.79	0.78	0.86
L2/3 (6962/5833)	0.95	0.95	0.95	0.99	0.89	0.90	0.88	0.96
L4 (3415/3103)	0.93	0.91	0.94	0.98	0.83	0.80	0.87	0.91
L5/6 (1710/1692)	0.93	0.93	0.93	0.98	0.78	0.77	0.80	0.86
L5/6-CC (2279/2106)	0.97	0.98	0.97	1.00	0.85	0.88	0.82	0.93
Microglia (1174/1321)	0.91	0.90	0.93	0.97	0.76	0.73	0.78	0.84
Neu-mat (1853/1679)	0.85	0.82	0.88	0.93	0.75	0.70	0.80	0.83
Neu-NRGN-I (321/268)	0.97	0.99	0.94	0.99	0.69	0.75	0.63	0.74
Neu-NRGN-II (828/631)	0.82	0.86	0.78	0.89	0.63	0.70	0.53	0.68
Oligodendrocytes (4587/7619)	0.83	0.86	0.81	0.91	0.77	0.79	0.75	0.85
OPC (5085/4562)	0.83	0.82	0.84	0.91	0.75	0.74	0.76	0.82

*The number of nuclei from ASD and controls are listed. ROC analysis was applied to obtain the AUC and the optimal cut-off point on the training set, and then the optimal cut-off was used to determine the predictive accuracy (ACC), sensitivity (SN), and specificity (SP) on the training and test sets.*

By examining the number of RFE genes in every cell type (Table 2), we found that in several cell types, such as AST-PP, IN-PV, L2/3, L4, and L5/6-CC, there are more RFE genes and the corresponding cell type-specific predictive models have better performances than other cell types (Figure 2A). This implies that these cell types may be more vulnerable in ASD and more genes may be dysregulated in these cell types. Then, for each cell type, we also applied edgeR (Robinson et al., 2010) to identify DE genes in ASD compared to controls. It can be seen that in the mentioned cell types above, there are indeed more DE genes, which also indicates that these cell types may be mainly affected by ASD. By performing hypergeometric tests, we found that the RFE genes are significantly overlapped with the DE genes identified by edgeR (Table 2). Then, we checked if building cell type-specific predictive models using edgeR genes would be better than the ones using RFE genes, while the model performances using RFE genes are better than the ones using edgeR genes (Supplementary File 1). This shows that genes that are not identified by edgeR may have predictive power for ASD. In addition, we also compared the RFE genes with the DE genes identified in the single-nucleus RNA-seq study of ASD (Velmeshev et al., 2019). We found that RFE genes are significantly overlapped with Velmeshev’s genes, especially for the cell types of microglia, L2/3, L4, and IN-VIP (Table 2). The model performances using RFE genes are significantly better than the ones using Velmeshev’s genes (Supplementary File 1), which may be because the number of Velmeshev’s genes is small. Next, we found that there are more SFARI ASD genes overlapped with RFE genes in neuron-related cell types. We also performed overrepresentation tests between RFE genes and SFARI ASD genes, and found that RFE genes are significantly overlapped with ASD genes (Table 2).

**TABLE 2:** The overrepresentation tests between RFE genes and differentially expressed genes identified by edgeR, differentially expressed genes identified in the study of Velmeshev et al. (2019), and SFARI ASD genes.

Cell type	Number of RFE genes	Overlapping genes/edgeR genes (FDR-adjusted *P*-value)	Overlapping genes/ASD genes (FDR-adjusted *P*-value)	Overlapping genes/Velmeshev’s genes (FDR-adjusted *P*-value)	Top five important genes
AST-FB	200	120/257 (1.5e−158)	22/299 (5.8e−09)	8/11 (1.4e−12)	***DPP10, TMSB4X, SPARCL1, ZFP36L1, PCDH9***
AST-PP	1000	667/1464 (0.0e+00)	98/299 (2.2e−34)	33/36 (2.1e−32)	*PTGDS, HSPA1A, TRPM3, RP11-179A16.1, *CIRBP
Endothelial	500	115/146 (3.2e−134)	40/299 (6.3e−11)	29/38 (1.5e−32)	***HERC2P3***, *AKAP12, ***TMSB4X*,** *RP11-649A16.1*, ***RPS28***
IN-PV	1000	384/695 (4.2e−251)	103/299 (2.7e−38)	14/14 (1.3e−15)	***AC105402.4***, ***MTATP6P1***, CNTNAP3, *CIRBP, *ARL17B*
IN-SST	1000	549/1346 (5.9e−291)	104/299 (5.3e−39)	16/17 (1.5e−16)	***SST, AC105402.4***, ***VGF***, ***HSPA1A, BCYRN1***
IN-SV2C	900	345/616 (7.4e−244)	100/299 (9.7e−40)	9/9 (1.1e−10)	***CCK***, ***BCYRN1***, ***AC105402.4*,*MEG3***, ***HSPB1***
IN-VIP	1000	676/1820 (0.0e+00)	104/299 (5.3e−39)	32/32 (7.1e−35)	***HSPA1A***, ***CCK***, *RPS15, ***MEG3***, *RGS12
L2/3	1000	863/4690 (7.8e−230)	107/299 (2.9e−41)	41/41 (2.0e−44)	***BCYRN1***, ***CCK***, *CNTNAP2, MEG3, *CAMK2N1
L4	1000	715/2477 (1.3e−294)	113/299 (3.7e−46)	40/42 (1.2e−40)	***BCYRN1, CCK***, *NCAM2, ***SLC17A7***, ***MTATP6P1***
L5/6	900	467/1069 (4.0e−281)	98/299 (2.7e−38)	5/5 (2.8e−06)	***BCYRN1, AC105402.4***, ***MTATP6P1***, ***ATP1B1***, ***SLC17A7***
L5/6-CC	1000	701/3183 (7.1e−202)	114/299 (7.5e−47)	7/7 (3.8e−08)	***BCYRN1***, ***CCK***, ***AC105402.4*,** *RP11-750B16.1*, ***MT-RNR2***
Microglia	200	74/106 (4.9e−112)	20/299 (1.4e−07)	38/49 (2.5e−58)	***FKBP5***, ***TMSB4X***, ***NEAT1***, ***SLC1A3***, ***CHN2***
Neu-mat	900	351/476 (1.7e−312)	116/299 (2.9e−53)	1/1 (7.5e−02)	***AC105402.4***, *XIST*, ***CAMK2N1***, ***MEG3***, ***ROBO2***
Neu-NRGN-I	100	2/2 (6.8e−05)	12/299 (7.0e−06)	4/6 (8.7e−08)	*RP11-750B16.1*, *PTMA, NRGN, GNAO1, TSPAN7
Neu-NRGN-II	100	7/8 (1.9e−14)	6/299 (3.8e−02)	2/4 (4.4e−04)	***PRNP***, ***NRGN***, ***STMN1***, ***RP11-750B16.1*** *PLP1*
Oligodendrocytes	600	410/1420 (9.4e−253)	57/299 (9.5e−19)	14/14 (1.2e−18)	*PTGDS, NRXN1, CNDP1, *ABCA2, ***CREB5***
OPC	900	528/1413 (1.9e−285)	102/299 (2.9e−41)	3/3 (4.4e−04)	***GPC5***, ***TMSB4X***, ***HSPH1***, *CNTNAP2, *OLIG1

The number of overlapping genes; the number of edgeR genes, Velmeshev’s genes, and ASD genes; and the FDR-adjusted hypergeometric test *P*-values are shown. The genes with top five importance are listed, of which edgeR genes are in boldface, SFARI ASD genes are underlined, and Velmeshev’s genes are marked with *.

For each cell type-specific predictive model built based on RFE genes, we calculated the importance of each RFE gene (Supplementary File 3). Table 2 lists the top RFE genes in each cell type. Figure 2B also demonstrates the expression of the top three RFE genes in ASD and control groups for the representative cell types, including AST-PP, endothelial, IN-PV, L2/3, microglia, oligodendrocytes, and OPC. The top genes among different cell types are distinct, implying the cell-type heterogeneity of ASD. However, some top genes appearing in several cell types are of note. For instance, gene *BCYRN1* (brain cytoplasmic RNA 1, a long non-coding RNA) has the largest importance in all excitatory neurons, including L2/3, L4, L5/6, and L5/6-CC. Gene *BCYRN1* is involved in the regulation of synaptogenesis, and there have been several literatures linking *BCYRN1* and Alzheimer’s disease, a neurological disease (Wan et al., 2017; Hu et al., 2018), which implies the possible association between *BCYRN1* and ASD. Besides, *BCYRN1* has been prioritized in a blood-based gene expression study of ASD (Ivanov et al., 2015).

To further characterize the cell-type heterogeneity of ASD, we compared the RFE genes across different cells. We performed gene ontology analyses using clusterProfiler (Yu et al., 2012), with background genes set as the genes in the analyzed gene expression matrix. The functions of cell type-specific RFE genes are different among different cell types (Supplementary File 4). For instance, in IN-PV, the enriched GO terms include neuron projection, axon, somatodendritic compartment, and cell part morphogenesis, while in L2/3, the top GO terms are associated with ribosome, cotranslational protein targeting to membrane, and protein localization to endoplasmic reticulum (Figure 2B).

### Cell Type-Specific Gene Sets Associated With ASD

**FIGURE 3:**
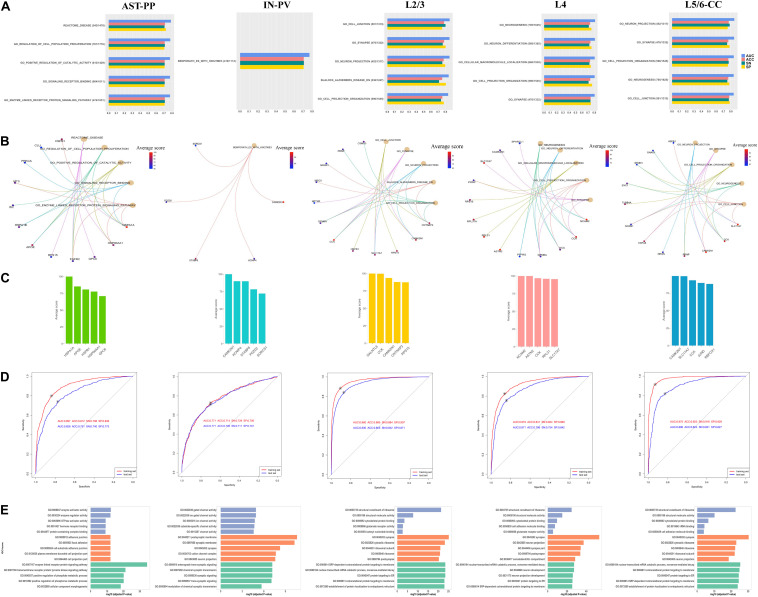
**(A)** The identified top five gene sets associated with ASD by constructing cell type-specific gene set-based predictive models. The number of overlapping genes between the gene expression data and the gene set, the total number of genes in the gene set, and the performances of corresponding cell type-specific gene set-based predictive models are shown. For each cell type, **(B)** illustrates the top five gene sets and the genes with top five importance in each gene set, and **(C)** plots the genes with top averaged importance. **(D)** The performances of predictive models built using genes with averaged importance >10% and **(E)** the enriched GO terms with these genes. The GO terms belonging to molecular functions, cellular component, and biological process are shown in blue, orange, and green, respectively.

In addition to screening individual genes associated with ASD, we also constructed cell type-specific gene set-based predictive models to screen ASD-related gene sets. For each cell type and each gene set, we extracted the expression level of the nuclei from the cell type in the genes included in the considered gene set, and constructed a predictive model (see section “Materials and Methods”). We retained the gene sets whose ACC, SN, and SP on test set are larger than 70%, and there are 5, 1, 88, 15, and 137 gene sets identified in cell types of AST-PP, IN-PV, L2/3, L4, and L5/6-CC, respectively (Supplementary File 5). Figure 3A shows the top five gene sets in each of these five cell types and the performances of corresponding cell type-specific gene set-based predictive models. For AST-PP, the top ASD-associated gene sets include *REACTOME_DISEASE, GO_REGULATION_OF_CELL_POPULATION_PROLIFERATI- ON, GO_POSITIVE_REGULATION_OF_CATALYTIC_ACTIVI- TY, GO_SIGNALING_RECEPTOR_BINDING*, and *GO_ENZY- ME_LINKED_RECEPTOR_PROTEIN_SIGNALING_PATHWAY*. For other neuron cell types, the ASD-associated gene sets are mostly related to cell junction, synapse, neuron projection, neurogenesis, neuron differentiation, and cell projection organization. By checking the top important genes in each cell type-specific gene set, we found that several genes appear in the majority of the gene sets; for example, gene *HSPA1A* [heat shock protein family A (*HSP70*) member 1A] shows up in all AST-PP specific ASD-associated gene sets (Figure 3B). Therefore, for each cell type, we analyzed the frequency of each gene included in the identified gene sets and calculated the averaged importance of genes (Supplementary File 5). Figure 3C shows the genes with top five averaged importance in each cell type. Gene *HSPA1A* is noted in AST-PP. Actually, heat shock proteins play a central role in the development of neurological disorders, of which *HSP70* family has been shown its functions (Turturici et al., 2011), and *HSPA1A*, a member of *HSP70* family, has already been associated with ASD (Lin et al., 2014). As to gene *CCK* (cholecystokinin), it is prioritized in excitatory neurons, which is a kind of gut peptide hormone. Gut peptide hormones have been found across different brain regions, and many of them are involved with ASD-related deficits (Qi and Zhang, 2020).

Next, based on the genes with averaged importance >10% in corresponding cell types, we re-constructed a cell type-specific predictive model for each of these five cell types. It is noted that their predictive performances are even better than the ones of the cell type-specific gene set-based predictive models (Figure 3D). We checked the functions of these genes (Supplementary File 6) and found that their functions are distinct, especially among AST-PP, IN-PV, and excitatory neurons (Figure 3E). In AST-PP, the top genes are associated with the functions of enzyme-linked receptor protein signaling pathway, transmembrane receptor protein tyrosine kinase signaling pathway, positive regulation of phosphorus and phosphate metabolic process, and cellular component morphogenesis. In IN-PV, the top genes are related to synaptic and postsynaptic membrane, cation channel complex, and neuron projection. As to the cell types of excitatory neurons, the top genes are associated with ribosome, SRP-dependent cotranslational protein targeting to membrane, nuclear-transcribed mRNA catabolic process, nonsense-mediated decay, and protein targeting to ER.

### A Multi-Label Classification Model Predicting Cell Type and Diagnosis

**FIGURE 4:**
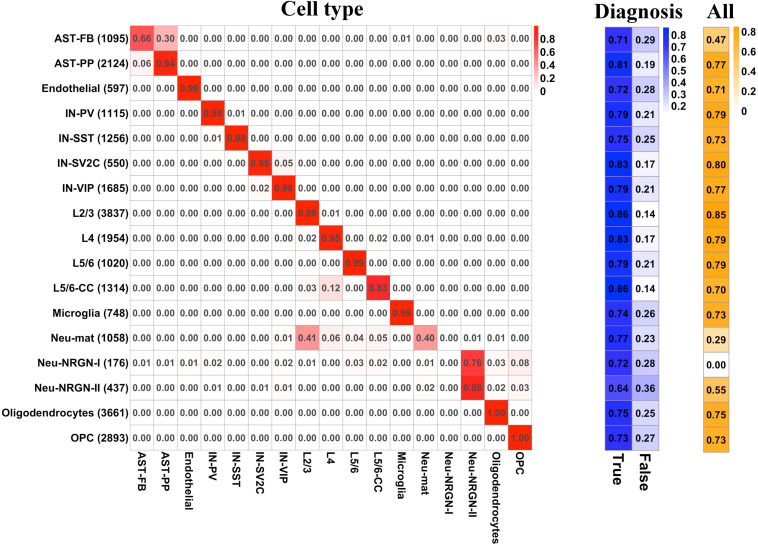
For each cell type, **(left)** the proportion of the number of nuclei predicted as each cell type to the total number of nuclei, **(middle)** the proportion of correct and incorrect predictions for the label of diagnosis, and **(right)** the proportion of correct predictions for all labels in the test set.

To predict the cell type and diagnosis of a given nucleus at the same time, we applied PLS to construct a multi-label predictive model (see section “Materials and Methods”). We split the whole gene expression data to a training set and a test set. For the diagnosis label, we extracted the predictive probability of training set and applied ROC analysis to obtain the optimal cut-off for determining the predictive diagnosis of each nucleus in training and test sets. For the cell type labels, the predictive cell type of each nucleus was set as the cell type whose predictive probability is the largest. The Hamming loss of the multi-label predictive model is 0.02, and the accuracy achieves 72.8 with 92.7% accuracy for cell-type labels and 78.5% accuracy for diagnosis label. Then, we examined the predictive performance of the model by cell type and by label. For each cell type, Figure 4 illustrates the proportion of the number of nuclei predicted as each cell type to the total number of nuclei, the proportion of correct and incorrect predictions for the label of diagnosis, and the proportion of correct predictions for all labels in the test set. It can be seen that for most cell types, the predictive cell types are correct, except for AST-FB, Neu-mat, and Neu-NRGN-I. Because AST-FB and AST-PP are cell clusters of astrocytes and they may have similar gene expression patterns, a part of nuclei from AST-FB is predicted as AST-PP. As both Neu-NRGN-I and Neu-NRGN-II are NRGN-expressing neurons, nuclei from Neu-NRGN-I were mostly predicted as Neu-NRGN-II. As to Neu-mat, more than 40% nuclei were predicted as L2/3, which may indicate that the gene expression patterns between Neu-mat and L2/3 are similar. For most cell types, the predictive accuracy of diagnosis label is larger than 70%, and the top highest accuracy values appear in L2/3, L5/6-CC, IN-SV2C, L4, and AST-PP, showing that these cell types may be more vulnerable in ASD.

## Discussion

Genetic studies have identified variants associated with ASD, while the causal variants and the specific cell types in which the disease-risk variants may be active are unclear. Genes may demonstrate diverse functions across different brain cell types. Different functions may be dysregulated and causal genes may be distinct across different brain cells in ASD. Recently, the newly available single-nucleus RNA-sequencing data of ASD (Velmeshev et al., 2019) makes it possible to study the cell-type heterogeneity of ASD directly. The authors identified DE genes between ASD and controls in a cell type-specific way and found that the top DE neuronal genes were identified in L2/3 and IN-VIP, and the top DE genes in non-neuronal cell types were identified in AST-PP and microglia. The relative changes of DE genes in L2/3 and microglia were the most predictive of clinical severity of ASD patients and the cell types that are recurrently affected across multiple patients included L2/3 and L5/6-CC. They concluded that synaptic signaling of upper-layer excitatory neurons and the molecular state of microglia are preferentially affected in ASD, and the dysregulation of specific groups of genes in cortico-cortical projection neurons correlates with clinical severity of ASD.

Actually, except for genetic and genomic studies, gene prioritization studies (Kong et al., 2012; Cogill and Wang, 2016; Guan et al., 2016; Oh et al., 2017) can be applied to detect ASD risk genes, which can help to identify high-confidence gene candidates. In this study, to characterize the cell-type heterogeneity of ASD and to identify cell type-specific genes and gene sets associated with ASD, we constructed multiple kinds of predictive models based on the human brain nucleus gene expression data of ASD and controls (Velmeshev et al., 2019). By constructing cell type-specific predictive models based on individual genes, we found that AST-PP, IN-PV, L2/3, L4, and L5/6-CC may be more vulnerable in ASD. They have more RFE genes and the corresponding cell type-specific predictive models have better performances. Actually, they have more DE genes identified by edgeR and more SFARI ASD genes. These indicate that more genes may be dysregulated in these cell types, and these cell types may be mainly affected by ASD. In addition, we also compared the RFE genes with the DE genes identified in the single-nucleus RNA-seq study of ASD (Velmeshev et al., 2019). We found that RFE genes are significantly overlapped with Velmeshev’s genes for all cell types, especially for microglia, L2/3, L4, and IN-VIP. The functions of genes with predictive power for ASD are different, and the top important genes are distinct across different cell types, highlighting the cell-type heterogeneity of ASD. However, some genes appearing as top important genes in several cell types are of note. For instance, gene *BCYRN1* has the largest importance in all excitatory neurons, including L2/3, L4, L5/6, and L5/6-CC. Gene *BCYRN1* is involved in the regulation of synaptogenesis, and there have been several literatures linking *BCYRN1* and Alzheimer’s disease, a neurological disease (Wan et al., 2017; Hu et al., 2018), which implies the possible association between *BCYRN1* and ASD. Besides, *BCYRN1* has been prioritized in a blood-based gene expression study of ASD (Ivanov et al., 2015).

As genes interact with others, the integrity of disease gene modules instead of individual genes may determine the manifestation of a disease in cells (Kitsak et al., 2016; Mohammadi et al., 2019). Therefore, in addition to identifying the individual cell type-specific risk genes, it is valuable to identify cell type-specific gene sets/modules associated with ASD. By constructing cell type-specific gene set-based predictive models, we also noted cell types of AST-PP, IN-PV, L2/3, L4, and L5/6-CC. The identified gene sets specific to these cell types are different. For AST-PP, the ASD-associated gene sets include *REACTOME_DISEASE, GO_REGULATION_OF_CELL_POPULATION_PROLIFERATI- ON, GO_POSITIVE_REGULATION_OF_CATALYTIC_ACTIVI- TY, GO_SIGNALING_RECEPTOR_BINDING*, and *GO_ENZY- ME_LINKED_RECEPTOR_PROTEIN_SIGNALING_PATHWAY*. For the other four neuronal cell types, the ASD-associated gene sets are mostly related to cell junction, synapse, neuron projection, neurogenesis, neuron differentiation, and cell projection organization. We found that gene *HSPA1A* appears as the most important gene in all AST-PP specific ASD-associated gene sets. Actually, heat shock proteins play a central role in the development of neurological disorders, of which the *HSP70* family has been shown its functions (Turturici et al., 2011), and *HSPA1A*, a member of *HSP70* family, has already been associated with ASD (Lin et al., 2014). Gene *CCK* is prioritized in L2/3, L4, and L5/6-CC, which is a kind of gut peptide hormone. Gut peptide hormones have been found across different brain regions, and many of them are involved with ASD-related deficits (Qi and Zhang, 2020).

Overall, we found that the functions of genes with predictive power for ASD are different and the top important genes are distinct across different cell types, depicting the cell-type heterogeneity of ASD. The findings suggest that L2/3, L4, L5/6-CC, AST-PP, and IN-PV are mainly affected in ASD. The results show that it may be feasible to use single cell/nucleus gene expression for ASD detection and the constructed predictive models can promote the diagnosis of ASD. Our method prioritizes ASD-associated cell type-specific genes and gene sets, which may be used as potential biomarkers of ASD, promoting the design of effective interventions.

## Data Availability Statement

The original contributions presented in the study are included in the article/Supplementary Material, further inquiries can be directed to the corresponding author/s. The codes were deposited on Github: https://github.com/JGuan-lab/Cell-type-specific-predictive-model.

## Author Contributions

JG conceived the study. YW, YL, and QY wrote the codes and analyzed the data. JG, YZ, and GJ interpreted the results. JG and YW wrote the manuscript. All authors approved the final manuscript.

## Conflict of Interest

YZ was employed by Xiamen YLZ Yihui Technology Co., Ltd. The remaining authors declare that the research was conducted in the absence of any commercial or financial relationships that could be construed as a potential conflict of interest.

## Supplementary Material

Supplementary Figure 1 The density plot of the percentage of variance explained by each factor across all genes **(A)** and highly variable genes **(B)**. Each curve denotes one factor.

Supplementary File 1 The performances of cell type-specific predictive models built based on all genes, top 500, 1000, and 1500 important genes, RFE genes, edgeR genes, and Velmeshev’s genes.

Supplementary File 2 The calculated gene importance in each cell type-specific predictive models built based on all genes.

Supplementary File 3 The calculated gene importance in each cell type-specific predictive models built based on RFE genes.

Supplementary File 4 The enriched GO terms with cell type-specific RFE genes.

Supplementary File 5 The identified ASD-associated cell type-specific gene sets along with their top five important genes and predictive performances. For these gene sets, the frequency and averaged importance of each gene included in the gene sets are listed.

Supplementary File 6 The enriched GO terms for genes with averaged importance >10% included in the identified ASD-associated cell type-specific gene sets.
