# Supplementary figures and images for: Cell Type-Specific Predictive Models Perform Prioritization of Genes and Gene Sets Associated With Autism
Source: Front Genet. 2021 Jan 15;11:628539. doi: 10.3389/fgene.2020.628539 (PMC7844401; doi:10.3389/fgene.2020.628539)

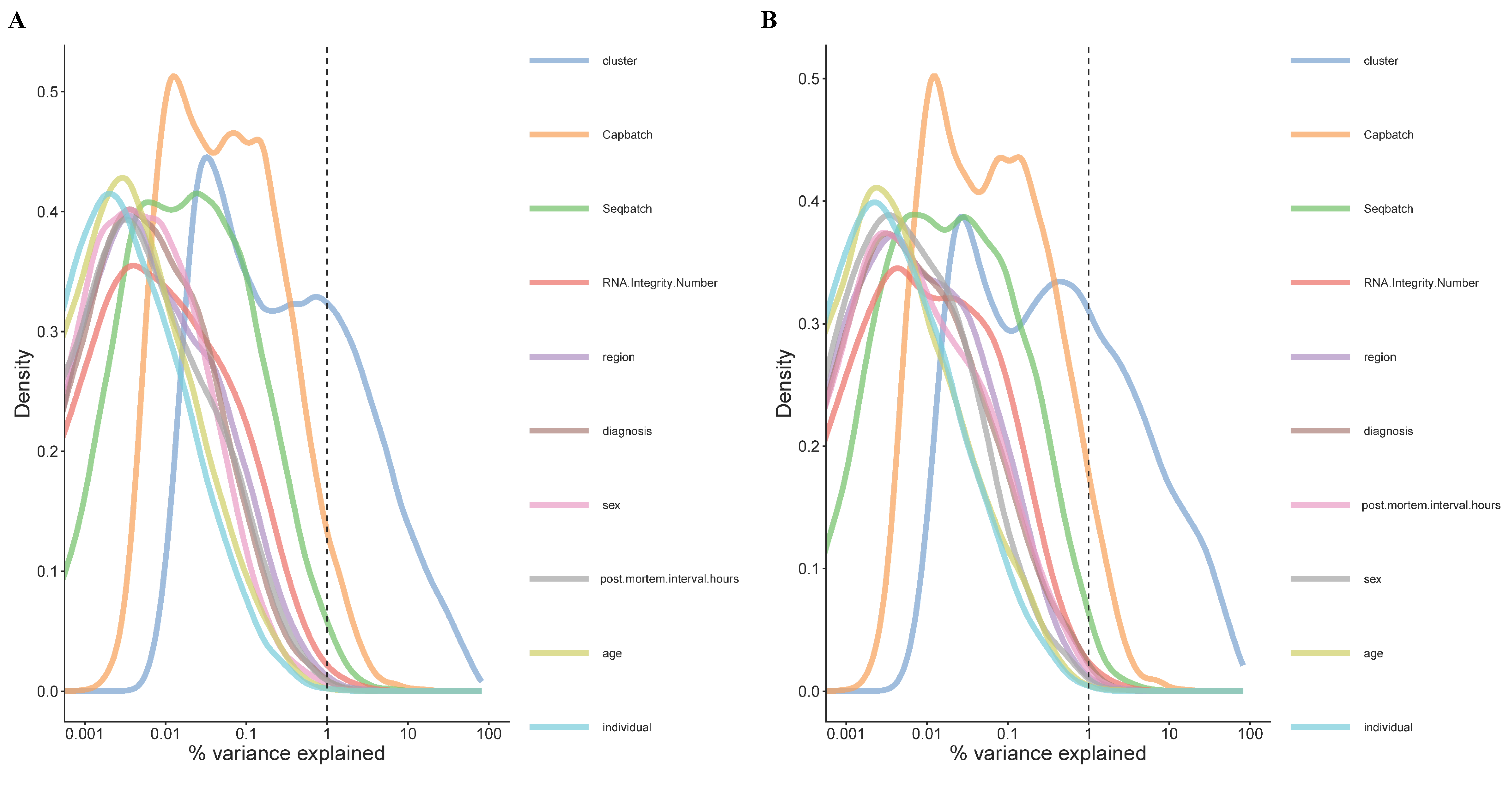

Supplement: Supplementary Figure 1 — The density plot of the percentage of variance explained by each factor across all genes (A) and highly variable genes (B). Each curve denotes one factor. [file Image_1.TIFF]
